# Posterior minimally invasive percutaneous short-segment fixation combined with mini-open anterior L5 corpectomy for metastatic tumor: a case report and literature review

**DOI:** 10.3389/fsurg.2026.1841067

**Published:** 2026-09-03

**Authors:** Talgat Kerimbayev, Meirzhan Oshayev, Murat Arlanbekov, Aizhan Baltabay, Serik Akshulakov

**Affiliations:** 1 Department of Spinal Surgery and Pathology of the Peripheral Nervous System, JSC “National Centre for Neurosurgery”, Astana, Kazakhstan; 2 Department of Pediatrics, CF “University Medical Center”, Astana, Kazakhstan; 3 JSC “National Centre for Neurosurgery”, Astana, Kazakhstan

**Keywords:** case report, corpectomy, hepatocellular carcinoma, L5 vertebral body, spinal metastasis

## Abstract

**Background:**

Metastatic tumors of the lumbar spine are a common complication of advanced malignancies. They often present with severe pain, neurological deficits, and spinal instability. Surgical removal of L5 vertebral body metastases is challenging due to the complex anatomy and biomechanical characteristics of the lumbosacral junction. Corpectomy using a combined approach is a common surgical strategy for achieving pain relief, adequate tumor resection, spinal cord and root decompression, and spinal stabilization. However, this approach may be associated with significant surgical trauma and blood loss. In this case report, we describe a surgical technique aimed at pain relief, mechanical stabilization, neurological preservation, and prevention of severe complications.

**Case description:**

A 62-year-old man with hepatocellular carcinoma presented with severe lumbar pain radiating to the left lower extremity, progressive lower limb weakness, and sensory deficits. Neurological examination revealed muscle strength of 3/5 in the lower extremities, Frankel grade C, and a Karnofsky Performance Status score of 40%. MRI and CT demonstrated metastatic destruction of the L5 vertebral body with spinal instability. The patient underwent a two-stage surgical procedure consisting of posterior minimally invasive percutaneous transpedicular fixation at the L4–S1 levels followed by mini-open anterior L5 corpectomy and anterior column reconstruction using a VLIFT titanium cage.

**Conclusion:**

The surgical procedure was performed without intraoperative complications. Total operative time was 4 h with an estimated blood loss of 800 mL. The patient was mobilized on the first postoperative day. Significant clinical improvement was observed: pain reduction from 8/10 to 2–3/10 on the Visual Analogue Scale and improvement in Karnofsky Performance Status from 40% to 80%. Postoperative CT confirmed adequate placement of the instrumentation and preservation of the posterior spinal column. Mini-open anterior L5 corpectomy combined with posterior minimally invasive percutaneous fixation may be a feasible surgical strategy for patients with L5 vertebral body metastasis. Preservation of the posterior spinal column may contribute to early mobilization, pain relief, and improved postoperative functional outcomes.

## 1 Introduction

Hepatocellular carcinoma is a malignant epithelial tumor showing hepatocytic differentiation (1). These tumors are often highly aggressive and present significant diagnostic and therapeutic challenges.

The skeletal system is one of the most common sites of metastasis for many types of solid tumors (2, 3). Bone metastases are associated with a high risk of serious complications such as pathological fractures, pain, hypercalcemia, and spinal cord compression, which can seriously impair patients’ quality of life (4, 5) and increase both morbidity and mortality (6, 7).

Spinal metastases occur in approximately 40%–70% of patients with malignant tumors (8). The L5 vertebra is the largest vertebra in the lumbar spine. A recent study conducted in China revealed that solitary L5 metastases constituted 10.89% of lower lumbar spine metastases (8). Additionally, it identified that pain was the chief presenting symptom among patients with lower lumbar spine metastases (8).

Corpectomy is indicated for the L5 vertebral body metastasis. Despite the potential for cure, corpectomy for L5 vertebral body tumors is a technically challenging procedure requiring multidisciplinary expertise and careful preoperative planning to limit potential morbidity and postoperative disability for the patient.

Selecting an appropriate surgical approach for L5 corpectomy is challenging due to the complex anatomy of the lumbosacral junction. The biomechanics of this region, marked by a sloped transition from the mobile lower lumbar spine to the relatively immobile sacrum and pelvis, subject surgical constructs to high stress from combined sliding and compressive forces (9). Therefore, careful selection of the surgical approach is essential.

A combined posterior-anterior approach is usually selected for the corpectomy for L5 tumors (10). Various complications of the posterior-anterior approach, such as longer operation time, more blood loss, and larger trauma, were reported by other studies (11, 12). To minimize the risk of these complications, several techniques were utilized in our case. Firstly, percutaneous transpedicular fixation was performed at the levels L4-S1 through a posterior minimally invasive approach: a small incision was made, avoiding extensive tissue trauma. Secondly, only the body of the L5 vertebra was resected through a mini-open anterior approach, and the lamina was left intact, which allowed us to preserve the integrity of the posterior column of the spine.

It is important to highlight that the number of case reports on the surgical removal of lower lumbar spine tumors is very limited. Among the studies included in our literature review, the specific combination of posterior minimally invasive percutaneous short-segment fixation and mini-open anterior L5 corpectomy with preservation of the L5 posterior column was not identified. Therefore, we decided to present a technically challenging case of a 62-year-old man who was diagnosed with hepatocellular carcinoma metastasis of the L5 vertebral body with primary origin in the liver, complicated by the malignant L5 nerve root compression. In addition, we describe in detail a two-stage technique - mini-open anterior approach to the resection of the body of L5 and its reconstruction, as well as a posterior minimally invasive approach to the posterior percutaneous short-segment fixation with preservation of the physiological and anatomical structures of the posterior column of the spine.

## 2 Case description

A 62-year-old patient was hospitalized at the JSC “National Center for Neurosurgery” with complaints of persistent pain in the lumbar spine, which increased with minor physical activity, bending, vertical positioning, and changing body posture (Table 1). The pain radiated to the left lower extremity along the distribution of the L5 nerve root. Additional symptoms included numbness in the left foot (hypesthesia), weakness in the lower extremities (Medical Research Council (MRC) Scale for Muscle Strength - grade 3/5), and a forced antalgic horizontal position of the body. These symptoms had persisted for two weeks. However, the patient reported lumbar pain for the preceding two years. The patient had a known diagnosis of hepatocellular carcinoma: cT3NxM1.

**Table 1:** Timeline of the patient supervision.

Date	Clinical event / Patient management
30.09.2025	Outpatient visit of the patient for persistent lumbar pain.
12.10.2025	MRI examination was performed.
30.10.2025	CT examination was performed.
02.11.2025	Multidisciplinary tumor board (neurosurgeons, oncologists, and radiologists): a consensus decision was made to proceed with surgical intervention as part of a palliative treatment strategy.
07.11.2025	The patient was admitted to the JNC “National Centre for Neurosurgery”.
08.11.2025	The patient was operated.
12.11.2025	Histology result was revealed.
14.11.2025	The patient was discharged from the hospital.
21.11.2025	Adjuvant spine SBRT was performed.
05.12.2025	A 4-week postoperative follow-up at the outpatient level was conducted.
27.01.2026	Death of the patient

**Figure 1:**
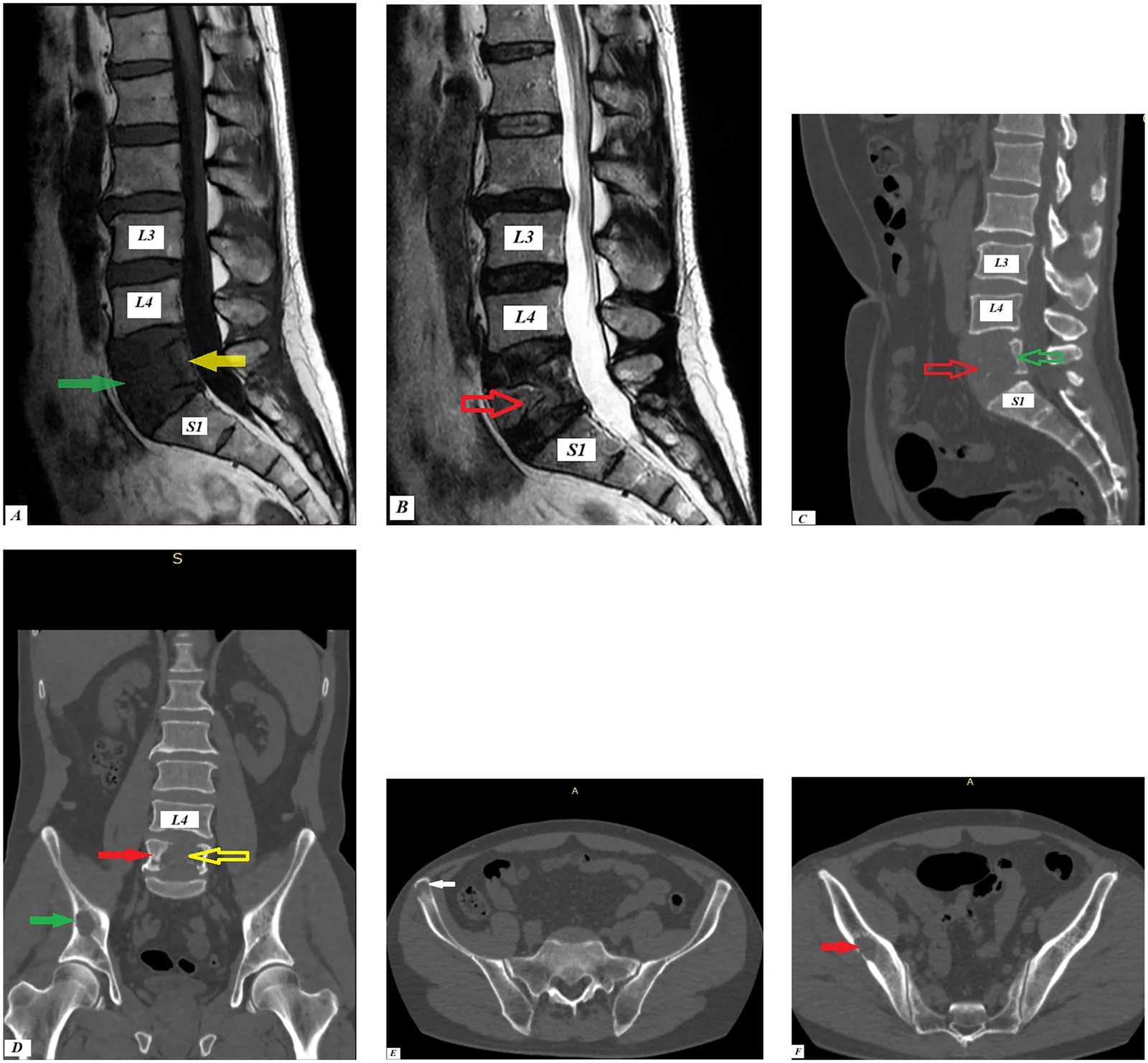
Lumbar spine MRI of the patient before surgery **(A,B)** and preoperative lumbar spine CT **(C–F)**. **(A)** Sagittal T1-weighted image. The green arrow indicates the L5 vertebra affected by hepatocellular carcinoma metastasis. The yellow arrow shows the residual part of the L5 vertebral body. **(B)** Sagittal T2-weighted image. The red arrow indicates involvement of the L5 vertebra by hepatocellular carcinoma metastasis. **(C)** Sagittal plane. The red arrow indicates a hepatocellular carcinoma metastasis with residual bony structures. The green arrow shows the posterolateral wall of the L5 vertebral body. **(D)** Coronal plane. The red arrow highlights the residual portion of the L5 vertebral body. The yellow arrow indicates a hepatocellular carcinoma metastasis. The green arrow marks a destructed area of the right iliac bone (metastasis). **(E)** Axial plane. The white arrow demonstrates the destructed area of the right iliac wing affected by a hepatocellular carcinoma metastasis. **(F)** Axial plane. The red arrow indicates another destructed area of the right iliac bone infiltrated by a hepatocellular carcinoma metastasis.

The Karnofsky Performance Status was assessed at 40%. The straight-leg raise test was positive on the left at 10 degrees. Pain intensity was rated 8/10 on the Visual Analogue Scale (VAS). Frankel’s grade was C. The MRI (Figures 1A, B) and CT (Figures 1C, D) of the lumbosacral region revealed L5 vertebral body metastasis and tumor metastasis in the right iliac bone (Figures 1E, F). No evidence of visceral metastases beyond the skeletal system was reported at the time of surgical decision-making. Weinstein, Boriani, and Biagini surgical staging (WBB) was 4–9, B-D. Tumor-related instability was unstable with 13 points based on the Spinal Instability Neoplastic Score system (SINS), suggesting the need for urgent surgical stabilization. Revised Tokuhashi Score was 7 points, indicating the mean survival of less than 6 months.

### 2.1 Pre-operation period

The patient underwent surgery 24 h after admission. Preoperative management included anti-edema therapy with dexamethasone (16 mg) and analgesic support. The basic therapy included antihypertensive medications: indapamide + perindopril tert-butylamine (2 mg + 0.625 mg) and bisoprolol 2.5 mg. Additionally, the patient received ademetionine 400 mg three times daily and ursodeoxycholic acid 250 mg.

### 2.2 Operation

The surgery was performed in two stages on the same day.

#### 2.2.1 Stage 1. Posterior paraspinal approach - percutaneous transpedicular fixation of L4-S1

The patient was placed in the prone position under general anesthesia. Antibiotic prophylaxis was administered with 2000mg of Ceftriaxone. The surgical field was disinfected with Betadine solution. Under fluoroscopic guidance, four cannulated screws were inserted percutaneously in a lateromedial trajectory through the pedicles into the vertebral bodies of L4 to S1. A transpedicular structure consisting of four screws, two rods, and four locking nuts was created.

#### 2.2.2 Stage 2. Anterior transabdominal, extraperitoneal approach – resection of the L5 vertebral body and interbody fusion using a VLIFT titanium cage

With the patient in the supine position, a cushion was placed beneath the sacrococcygeal spine to facilitate access. The surgical level was determined using an image intensifier. After preparing the surgical field with Betadine solution, a transverse skin incision of up to 7.0 cm was made at the junction of the upper and middle thirds of the distance between the biiliac line and the superior border of the pubic symphysis.

The anterior linea alba was mobilized and dissected longitudinally. Using a combination of blunt and sharp dissection, the aponeurosis was separated bilaterally from the underlying rectus abdominis muscles. From under the muscle and orienting toward the lateral iliac fossa, the fibers of the transversalis fascia were bluntly separated to access the retroperitoneal space.

A Triplex system was inserted. The surgical level was then reconfirmed using an image intensifier. A surgical microscope was used for enhanced visualization. The right and left common iliac arteries and veins were carefully mobilized at the level of the L4–S1 discs. The intra-abdominal fascia and surrounding fatty tissue were bluntly dissected until the anterior longitudinal ligament of the spine was exposed. The anterior longitudinal ligament was then dissected. Curettage of the L4–L5 and L5–S1 intervertebral discs was performed to prepare the site for interbody fusion.

Using a diamond-tipped electric burr, a conochotome, and Kerisson rongeurs, the L5 vertebral body was resected. During the procedure, a sharp decrease in bone density was observed, with partial replacement by soft tissue. Profuse bleeding from the vertebral body was observed. A complete blood count and a biochemical blood test were taken intraoperatively - anemia was identified, and reinfusion therapy and albumin transfusion were started. Fragments of the resected vertebra were sent for histopathological examination. Following the resection of the L5 vertebral body, pulsation of the dura mater improved. A VLIFT titanium cage was installed in the L4-S1 interbody space. Image intensifier monitoring confirmed satisfactory positioning and condition of the VLIFT titanium cage and associated posterior metal structure. The amount of blood loss during the operation was 800 mL. The duration of operation was 4 h.

### 2.3 Histology

In the examined histological specimens stained with hematoxylin and eosin, the tumor fragments were composed of proliferations of multiple acinar structures. These structures varied in shape and size and were lined by polymorphic, hyperchromatic cells. Tubular and cribriform patterns were also observed. Within the fibrous stroma, tumor cells formed scirrhous structures. Despite these atypical histological features, a diagnosis of poorly differentiated hepatocellular carcinoma metastasis was established based on immunohistochemical findings confirming hepatocellular differentiation.

### 2.4 Postoperative period and follow-up

The patient was mobilized on the first postoperative day. Neurological status remained at the preoperative level throughout the postoperative period, and pelvic functions were preserved. At discharge, the patient was able to ambulate independently. The Karnofsky Performance Status was 80%. VAS score decreased significantly to 2–3 points.

**Figure 2:**
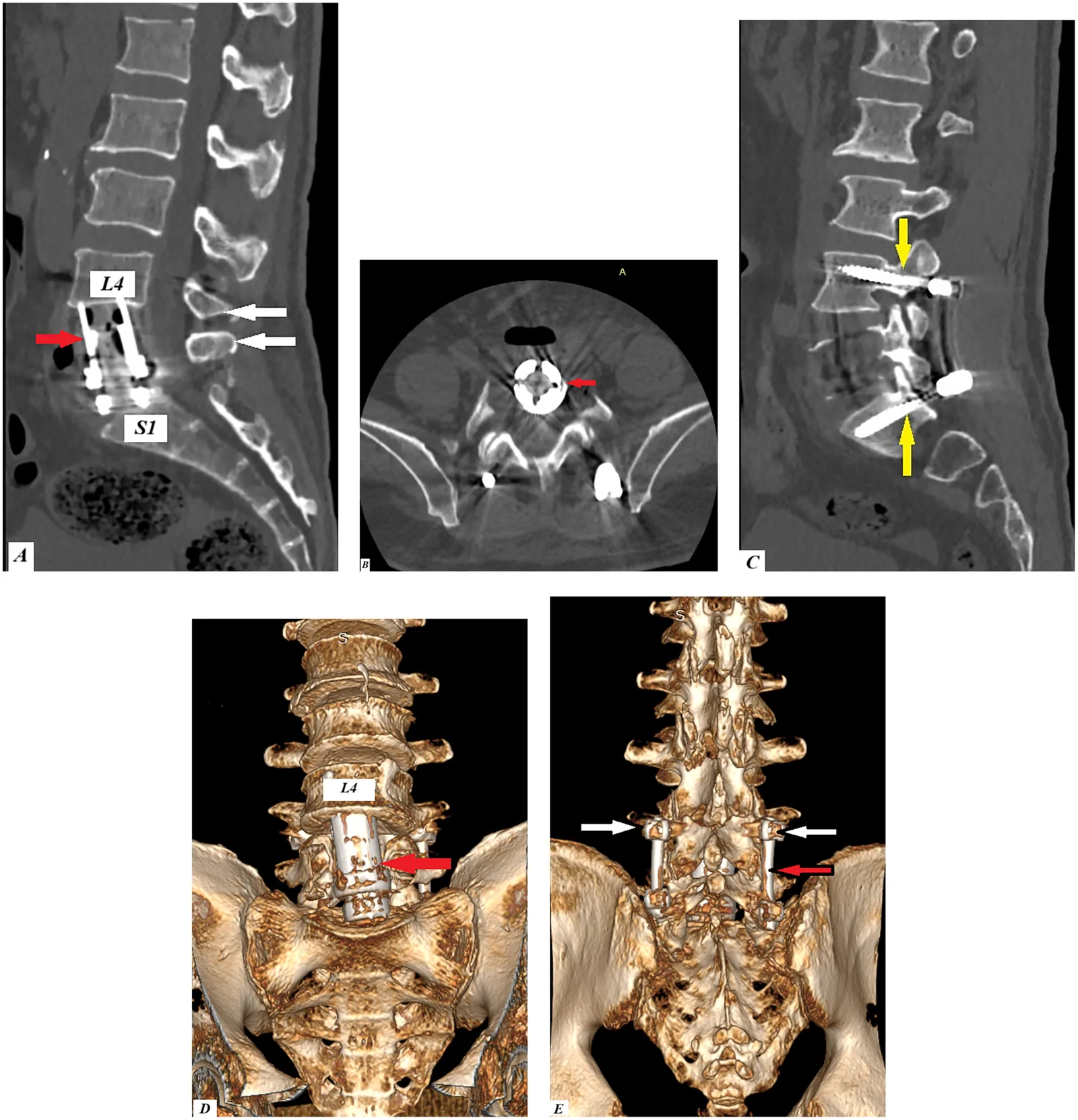
Postoperative lumbar spine CT images **(A–C)** and 3D reconstructions **(D,E)**. **(A)** Sagittal plane. The red arrow indicates the VLIFT titanium cage placed instead of the resected L5 vertebra. The white arrows point to the spinous processes of L4 and L5, confirming preservation of the posterior column. **(B)** Axial plane. The red arrow shows the centrally positioned VLIFT titanium cage. **(C)** Sagittal plane. The yellow arrows indicate cannulated screws inserted into the pedicles of L4 and S1. **(D)** Anterior 3D reconstruction. Total resection of the L5 vertebral body is visualized. The red arrow highlights the VLIFT titanium cage. **(E)** Posterior 3D reconstruction. The posterior column is completely preserved. The white arrows show the pedicle screws of L4, and the red arrow indicates the connecting rod.

A control CT scan of the lumbar spine was performed in the postoperative period (Figures 2A–E). The patient was discharged from the department on the 7th day after the operation and referred to stereotactic radiosurgery for further treatment due to resistance to regular radiotherapy (13).

Two weeks after surgery, the patient underwent a single course of postoperative adjuvant spine SBRT, delivered in two fractions. A 4-week postoperative follow-up at the outpatient level revealed the following findings: spinal pain, neurological deficits, and clinical evidence of implant-related complications were not observed. The patient was able to walk independently. No postoperative complications were observed from discharge through the 11-week follow-up period. A contrast-enhanced MRI of the lumbosacral spine was scheduled for the three-month follow-up. However, the patient died 11 weeks after the operation due to progression of hepatocellular carcinoma before the planned examination.

## 3 Discussion

Metastatic carcinoma of the spine is the most common form of malignant bone disease (14). Tumors that frequently metastasize to the spine include breast, lung, kidney, liver, prostate, and thyroid carcinomas, as well as melanoma, multiple myeloma, lymphoma, and colorectal cancer (15). The L5 vertebra poses surgical challenges due to its anatomical position at the lumbosacral junction and its exposure to biomechanical load. For this reason, surgical treatment of tumors at this level requires careful planning to achieve pain relief, adequate decompression, and maintain spinal stability.

Several surgical approaches for the treatment of L5 vertebral body tumors were described in the recent literature, including posterior-only, anterior-only, and combined posterior- anterior techniques. Posterior-only approach allows adequate tumor removal in certain cases; however, they often require extensive muscle dissection and multilevel instrumentation, which may increase surgical morbidity (16). Conversely, combined approach provides improved access to the anterior spinal column (17). It also facilitates effective decompression and reconstruction (17). However, combined approaches are associated with longer operation duration and increased intraoperative blood loss (11, 12).

The patient in our case had the diagnosis of hepatocellular carcinoma, with metastasis to the spine. A decision of a combined approach consisting of posterior minimally invasive percutaneous fixation followed by mini-open anterior L5 corpectomy was made by our team of neurosurgeons. The decision-making process was consistent with the NOMS framework: four fundamental assessments were made within it (27). Neurologically, the patient’s progressive motor deficit, severe pain (VAS = 8/10), and L5 nerve-root compression required direct decompression. Oncologically, surgery was not intended to achieve curative resection or oncological margins, and postoperative radiation remained part of the multidisciplinary treatment strategy. Mechanically, extensive destruction of the L5 vertebral body and a SINS score of 13 indicated instability that could not be adequately managed with radiotherapy alone. Thus, it required anterior-column reconstruction and posterior fixation. Systemically, the presence of metastatic disease (M1), a revised Tokuhashi score of 7, limited estimated survival, and poor preoperative functional status supported a surgical strategy aimed at rapid pain relief, stabilization, and early mobilization. For this reason, the purpose of surgery was decompressing the neural structures and restoring mechanical stability by the minimum intervention. The use of percutaneous short-segment fixation and a mini-open anterior approach was intended to reduce surgical morbidity while achieving these palliative objectives.

According to previous studies (12, 18–21), posterior-only and combined approaches involving large muscle dissections and multilevel instrumentation are associated with increased blood loss and prolonged recovery. A systematic review, analyzing articles on corpectomy and spondylectomy of thoracolumbar metastasis, demonstrated that approaches that require posterior muscle dissection and significant bone work lead to more blood loss (22), which may reduce survival in oncological patients (23). In contrast, posterior minimally invasive percutaneous fixation followed by mini-open anterior corpectomy seems to reduce tissue trauma, decrease intraoperative bleeding, and facilitate earlier postoperative mobilization. In our case, total blood loss was 800 mL, which is lower compared to the volumes reported in several studies included in our literature review (12, 18–21).

Another important step in the surgical treatment of the L5 vertebral body metastasis was the reconstruction of anterior column. In our case, anterior corpectomy contributed to sufficient decompression and immediate stabilization of the spinal column. Also, placement of the VLIFT titanium cage anteriorly maintained spinal alignment, provided adequate restoration of vertebral height, and stable support for the lumbosacral junction.

Short-segment fixation at L4–S1 may be associated with increased mechanical stress at the lumbosacral junction; however, in our case it was considered sufficient given the patient’s limited life expectancy.

Consistent with the WFNS Spine Committee recommendations, selection of the surgical strategy was based not solely on the SINS score but also on neurological status, tumor characteristics, systemic condition, functional status, and estimated survival (28).

Vertebroplasty with open decompression was considered during multidisciplinary planning. However, it was not selected because the patient had extensive destruction of the L5 vertebral body, disruption of the posterior vertebral wall, neural compression, and marked mechanical instability (SINS 13). Therefore, vertebroplasty has an increased risk of cement leakage into the spinal canal (24) and was not considered to provide sufficient restoration of anterior-column load-bearing capacity. Furthermore, open posterior decompression would require disruption of the posterior osseoligamentous structures, whereas preservation of the posterior column was one of the objectives of the selected approach.

Separation surgery combined with short-segment minimally invasive fixation and subsequent stereotactic radiotherapy was also considered. However, the main surgical problem was not limited to neural decompression. Extensive destruction of the L5 vertebral body had resulted in substantial loss of anterior-column support at the mechanically demanding lumbosacral junction. Therefore, separation surgery and posterior fixation alone were considered insufficient to provide immediate and reliable reconstruction of the anterior column. Anterior corpectomy and cage reconstruction were selected to restore load-bearing capacity, relieve neural compression, and permit early mobilization.

There are several advantages of a posterior minimally invasive fixation with mini-open anterior corpectomy. These are reduced surgical trauma, preservation of posterior spinal column, effective decompression, prevention of spinal instability, and early postoperative mobilization. The patient in our case was able to ambulate independently on the first postoperative day and demonstrated significant improvement in pain and functional status. Additionally, the patient reported significant improvement in quality of life and pain control after surgery.

Nevertheless, our study has several limitations. First, it describes a single clinical case, which limits the generalizability of the findings. Second, follow-up was limited to 11 weeks because the patient died from progression of hepatocellular carcinoma before the planned 3-month MRI examination. Consequently, long-term local tumor control, fusion, cage position, and construct durability could not be assessed. Third, postoperative improvement in quality of life was not evaluated using a validated patient-reported outcome measure. We recommend conducting research with larger patient cohorts to evaluate the effectiveness of this surgical treatment method.

A literature review was conducted by our team on the surgical removal of the lower lumbar spine tumors (Table 2). A literature search was carried out of two electronic databases: Medline via Ovid and Pubmed for relevant studies published between 2000 and 2025. The main search terms involved “spinal cord tumors”, “lumbar vertebrae”, “corpectomy”, “spondylectomy”, and “surgical resection”. Case studies and case series examining adult patients who were surgically treated for lower lumbar spine tumors were included in the review. The other types of studies, studies investigating children, studies analyzing cervical, thoracic, upper lumbar, and sacral spine tumors, and studies investigating traumatic and degenerative spine disorders were excluded.

**Table 2:** Characteristics of the studies identified by a literature review.

Author, year	Study type	Number of patients	Tumor type	Tumor location	Surgical approach	Surgery technique	Posterior column preservation	Amount of blood loss	Duration of operation	Clinical outcome
Kawahara, 2011 (18)	Case series	10	1 aneurysmal bone cyst; 4 giant cell tumors; 2 breast cancer metastases; 1 renal cell carcinoma metastasis; 1 liposarcoma metastasis; 1 symptomatic hemangioma	L4 and L5	Combined posterior-anterior	*Three stages. Posterior approach:* en block laminectomy; removal of the posterior halves of the anterior column at the craniocaudal adjacent levels of the lumbar tumor. *Anterior approach:* en block corpectomy, anterior spinal reconstruction. *Posterior approach:* Adjustment of the posterior instrumentation; application of the connector between the posterior rods and anterior spacer if 2 or 3 vertebrae were removed.	Posterior column was resected	mL: 1) 6540/4645 2) 12 370 3) 3920 4) 11 290/2170 5) 500/400 6) 2560 7) 1630 8) 2540/680 9) 2000/ 390 10) 1800/290	Min: 1) 590/660 2) 1205 3) 800 4) 465/525 5) 545/435 6) 815 7) 890 8) 660/660 9) 679/600 10) 650/560	7 no evidence of disease; 1 alive with disease; 1 death of disease; 1 death of another disease
Santiago-Dieppa, 2014 (25)	Case study	1	Enneking stage III giant cell tumor	L4 and L5	Combined posterior-anterior	*Two stages.* *Posterior approach:* L3 laminectomy, medial L3–L4 facetectomy, *en bloc* removal of the L4 and L5 lamina, bilateral resection of the L3–L4 and L4–L5 facet joints, bilateral removal of the pedicles at L4 and L5, partial discectomies at L3–L4 and L5–S1, cross-link was placed at the L4–L5 level, Songer cables were passed anteriorly along the L4. *Anterior approach:* Anterior lumbosacral reconstruction using a VLIFT titanium cage at L3-S1. Two Songer cables were fastened to the cage. An anterior tension band (ATB) plate that spanned from L3 to S1 was installed. A polytetrafluoroethylene graft was placed between major vessels and the instrumentation.	Posterior column was resected	N/A	N/A	No evidence of tumor recurrence or instrumentation failure at more than 2 years of follow-up
Shousha, 2014 (19)	Case series	11	3 kidney cancer metastases, 1 lung cancer metastases, 1 thyroid cancer metastasis, 3 plasmacytoma, 1 lymphoma, 1 chordoma, 1 aneurysmal bone cyst.	L5	Posterior approach in one patient due to timorous infiltration of the L5 root, Combined approach in the rest 10 patients	*Posterior-only approach:* procedure was done with meticulous decompression of the nerve root followed by implantation of a narrow cage from posterior to anterior and fixation with pedicular screws L4–S1. *Combined approach:* *A standard left-sided anterolateral retroperitoneal approach:* Mobilization of distal aorta and the common iliac vessels, dissection of L4/L5 disk to the left common iliac vessels, exposure of L5/S1 disk underneath the bifurcation, complete resection of these 2 disks, L5 corpectomy, decompression of spinal canal, placement of titanium cage filled with bone cement in the corpectomy defect. *Posterior approach:* traditional posterior surgery with pedicle screws and exposure of the posterior elements, posterior stabilization in L4-S1, insertion of short pedicular screws in L5 pedicles to enhance the stability.	Posterior column was preserved in 10 cases, data on preservation was not reported in one case	Mean amount – 2.9 L	Mean time – 140 min	3 died of disease, 2 alive with disease, 6 no evidence of disease
Huang, 2018 (12)	Case series	9	2 breast cancer metastases, 2 thyroid cancer metastases, 1 prostate cancer metastasis, 1 hepatocellular carcinoma metastasis, 1 endometrial cancer metastasis, 1 non-small cell lung cancer metastasis, 1 renal cell carcinoma metastasis.	L4	Posterior-only approach	*Step 1:* a vertical incision over spinous process, instrumental fixation to maintain spinal stability at least 2 levels above and below the involved vertebra. *Step 2:* laminectomy at the adjacent levels above and below the lesion, removal of superior and inferior articular processes of the immediate neighboring vertebra, passing of T-saw guide and threadwire saws, cutting of bilateral pedicles, en block removal of entire posterior elements of the involved vertebra, dissection of the dural sac. *Step 3:* mobilization of bilateral nerve roots of L3 and L4 to the lumbosacral plexus nerves, placement of 2 T-saws in the L3-L4 and L4-L5 disc spaces, complete discectomy. *Step 4:* anterior reconstruction of L4 with a titanium mesh cage or VLIFT expandable Cage filled with autogenous bone, allograft bone, or bone cement; compression of a posterior instrumentation; placement of a drain.	Posterior column was resected	mL: 1) 1900 2) 3000 3) 3000 4) 1940 5) 2100 6) 3000 7) 2050 8) 2500 9) 2300	Min: 1) 245 2) 280 3) 270 4) 255 5) 265 6) 290 7) 300 8) 320 9) 310	3 died of disease, 5 no evidence of disease, 1 alive with disease
Li, 2019 (20)	Case study	1	Breast cancer metastasis	L5	Posterior-only approach	Midline incision from L3 to S1 over spinous process, implantation of 6 suit pedicle screws into L3/4 and S1, exposition of posterior elements of L5, removal of caudal part of the spinous process and lamina of the L4, removal of posterior spinous process, lamina and pedicles of L5 as one piece with an osteotome, dissection and protection of L4/5 nerves and dura mater, exposition of L4/5 and L5/S1 intervertebral discs with an osteotome, anterior reconstruction with a titanium mesh cage filled with autogenous iliac bones, compression of the rod-screws through L4 to S1.	Posterior column was resected	9000 mL	10 h	Death of another disease
Yang, 2019 (21)	Case series	7	3 giant cell tumor, 1 gastrointestinal metastasis, 1 thyroid cancer metastasis, 1 neurofibroma, 1 chondrosarcoma	L5	Posterior –only approach	Midline incision from L3 to S2, placement of pedicle screws into L3/4 and S1/2, decompression, retraction of paraspinal tissues, removal of caudal part of the L4 spinous process and lamina, removal of the posterior spinous process, lamina and pedicles of L5 as a whole with osteotome, dissection and protection of the dural sac and L4/5 nerves. Fabrication of autonomous iliac graft, dissection between the prevertebral tissues and the vertebra from L4/5 to L5/S1 intervertebral disc, fixation of a feasible rod to the contrary side of the iliac graft, cutting the above two vertebral discs, corresponding posterior and anterior longitudinal ligament with an osteotome, TES of L5. Anterior reconstruction with the titanium mesh cage cylinder or expandable artificial vertebra filled with allogenic bone. After completion of reconstruction the iliac graft was fixed back.	Posterior column was resected	mL: 1) 3500 2) 2000 3) 2000 4) 4000 5) 1500 6) 1600 7) 3000	Min: 1) 390 2) 445 3) 290 4) 380 5) 320 6) 335 7) 400	4 no evidence of disease, 2 deaths of disease, 1 alive with a newly diagnosed metastasis

Six eligible studies were identified: four of them were case series (12, 18, 19, 21), and two were case studies (20, 25). The number of patients in the case series varied from 7 to 11. Tumors were located in the L4 in one study (12), in the L5 in three studies (19–21), and in both L4 and L5 in two studies (18, 25). The giant cell tumor and breast cancer metastasis were the common types of cancer among patients included in six studies. It is important to mention that the surgical procedure performed in our case does not represent an *en bloc* resection but rather a palliative corpectomy.

Three studies utilized a posterior-only approach (12, 20, 21), two combined posterior-anterior approach (18, 25), and one used both posterior-only and combined posterior-anterior approach (19). The posterior column was resected in five studies involving total *en bloc* spondylectomy (12, 18, 20, 21, 25). In Shousha et al. (19), it was preserved in the 10 patients treated using an anterior L5 corpectomy followed by posterior pedicle-screw fixation; preservation of the posterior column could not be determined in the single posterior-only case because posterior-element management was insufficiently described. A reconstruction of the vertebral body was carried out using a titanium cage (18, 19), a titanium mesh cage (12, 20, 21), a VLIFT titanium cage (12, 25), and an artificial vertebra (21). These constructions were filled with autogenous bone (12, 18, 20), allogenic bone (12, 21), and bone cement (12, 19). The amount of blood loss during the operation varied from 900 mL to 13460 mL. The duration of operation ranged between 140 and 1320 min.

14 patients demonstrated no evidence of disease, whereas 4 patients died of disease in studies that examined the combined approach. On the other hand, 9 patients showed no evidence of disease, and 5 died of disease in studies that explored the posterior-only approach. However, it is important to mention that the cases differed widely from one another. The essential factors such as gender, age, tumor type, and number of metastases which affect the outcome varied substantially among cases. For this reason, a great contrast in the operation time (140 vs. 1320 min) and amount of blood lost (900 vs. 13460 mL) can be observed, and no definitive conclusion can be drawn due to heterogeneity of the studies.

The surgical approach and surgery technique should be selected individually, considering the patient’s condition, their will, functional status, tumor type, number of metastases, life expectancy, and surgeon’s experience (26).

## 4 Conclusion

After careful multidisciplinary evaluation and discussion of our case, L5 corpectomy with posterior minimally invasive percutaneous short-segment transpedicular fixation may be a feasible technique for pain relief, mechanical stabilization, and preservation of neurological function. In conclusion, preservation of the posterior column was safe and effective in this case, enabling early mobilisation and improved functional recovery.

## Data Availability

The original contributions presented in the study are included in the article/Supplementary Material, further inquiries can be directed to the corresponding authors.
